# Histone modification alteration coordinated with acquisition of promoter DNA methylation during Epstein-Barr virus infection

**DOI:** 10.18632/oncotarget.19423

**Published:** 2017-07-21

**Authors:** Sayaka Funata, Keisuke Matsusaka, Ryota Yamanaka, Shogo Yamamoto, Atsushi Okabe, Masaki Fukuyo, Hiroyuki Aburatani, Masashi Fukayama, Atsushi Kaneda

**Affiliations:** ^1^ Department of Molecular Oncology, Graduate School of Medicine, Chiba University, Chiba, Japan; ^2^ Department of Pathology, Graduate School of Medicine, The University of Tokyo, Tokyo, Japan; ^3^ Genome Science Division, Research Center for Advanced Science and Technology, The University of Tokyo, Tokyo, Japan

**Keywords:** DNA methylation, Epstein-Barr virus, gastric cancer

## Abstract

Aberrant DNA hypermethylation is a major epigenetic mechanism to inactivate tumor suppressor genes in cancer. Epstein-Barr virus positive gastric cancer is the most frequently hypermethylated tumor among human malignancies. Herein, we performed comprehensive analysis of epigenomic alteration during EBV infection, by Infinium HumanMethylation 450K BeadChip for DNA methylation and ChIP-sequencing for histone modification alteration during EBV infection into gastric cancer cell line MKN7. Among 7,775 genes with increased DNA methylation in promoter regions, roughly half were “DNA methylation-sensitive” genes, which acquired DNA methylation in the whole promoter regions and thus were repressed. These included anti-oncogenic genes, e.g. *CDKN2A*. The other half were “DNA methylation-resistant” genes, where DNA methylation is acquired in the surrounding of promoter regions, but unmethylated status is protected in the vicinity of transcription start site. These genes thereby retained gene expression, and included DNA repair genes. Histone modification was altered dynamically and coordinately with DNA methylation alteration. DNA methylation-sensitive genes significantly correlated with loss of H3K27me3 pre-marks or decrease of active histone marks, H3K4me3 and H3K27ac. Apoptosis-related genes were significantly enriched in these epigenetically repressed genes. Gain of active histone marks significantly correlated with DNA methylation-resistant genes. Genes related to mitotic cell cycle and DNA repair were significantly enriched in these epigenetically activated genes. Our data show that orchestrated epigenetic alterations are important in gene regulation during EBV infection, and histone modification status in promoter regions significantly associated with acquisition of *de novo* DNA methylation or protection of unmethylated status at transcription start site.

## INTRODUCTION

Cancer development and progression occur through accumulation of genetic and epigenetic disruptions [1, 2]. Almost 70% of annotated gene promoters have CpG-rich sequences called “CpG islands”, which are concentrated with non-methylated CpGs in somatic cells. DNA methylation of promoter CpG islands correlates with silencing of the downstream genes. Many tumor suppressor genes were *de novo* methylated and inactivated in cancer cells, which contributes to carcinogenesis [2, 3]. These aberrant DNA methylations seemed to occur in a non-random manner, and many types of cancer are classified into several epigenotypes based on the degree of DNA methylation. Gastric cancer is classified into at least three DNA methylation phenotypes: low-methylation, high-methylation, and extensively high-methylation epigenotypes [4]. These methylation epigenotypes reflect molecular subtypes such as microsatellite-instable (MSI) gastric cancer or Epstein-Barr virus (EBV)-associated gastric cancer [5, 6].

As the standpoint of the cause for aberrant DNA methylation in gastric cancer, EBV is demonstrated to induce *de novo* DNA methylation in host gastric epithelial cells during infection, which may be crucial for EBV-positive gastric carcinogenesis [4, 7]. EBV is a γ-herpes virus establishing lifelong asymptomatic infection called “latency” in B-lymphocytes in almost all human populations. The latency is restricted by the host cell types, and EBV is detected in many types of tumor tissues, not only in lymphoid malignancies, but also in many epithelial tumors [8]. Almost 10% of gastric cancer cases are EBV-positive [9]. In gastric cancer cells, EBV forms type I latency, where viral genome undergoes dense DNA methylation compared to other latencies, and the host genome shows extensively high-methylation epigenotype [4], named EBV-CIMP [6]. EBV infection itself was shown to establish this extensive DNA methylation in our previous study [4, 7].

Another epigenetic process, histone modification, which is a post-translational modification of the histone tails, mostly occurs at lysine and arginine residues. The high levels of H3K4 trimethylation (H3K4me3) or H3K27 acetylation (H3K27ac) at the promoters are implicated in the transcriptional activation, whereas H3K27 trimethylation (H3K27me3) is correlated with gene repression and silencing [10]. The establishment of appropriate histone modification patterns are essential for the normal development and tissue differentiation, while mutations of these modulators and aberrations of histone modifications are associated with cancer development [11, 12]. Several gene specific or genome-wide studies of histone modification alterations have been reported in gastric cancer [13–15]. However, alterations of EBV-positive gastric cancer and their association with aberrant DNA methylation are mostly unknown.

To clarify epigenomic alteration in promotor regions of host cell during EBV infection, we analyzed DNA methylation using Infinium HumanMethylation 450K BeadChip (Illumina, San Diego, CA, USA) and alteration of histone modifications, e.g., H3K4me3, H3K27ac, and H3K27me3, by ChIP-sequencing using *in vitro* system of EBV infection into gastric cancer cell line MKN7. A total of 9,275 genes presented newly methylated CpG probes of Infinium BeadChip in promoter regions, which are regarded as DNA methylation-induced genes. These DNA methylation-induced genes were divided into two gene types. Roughly half of the DNA methylation-induced genes were “DNA methylation-sensitive” genes which acquired DNA methylation in whole promoter CpG probes, and the other half represented “DNA methylation-resistant” genes. DNA methylation-resistant genes showed continuous non-methylated CpGs at immediate vicinity of the transcription start site (TSS) and maintained gene expression as if the TSS was protected from the DNA methylation-induction pressure. Histone modifications were also dynamically altered at promoter regions. The activated genes were related to mitotic cell cycle and DNA repair, while inactivated genes were related to apoptosis. It seemed that the protection of unmethylated TSS status at DNA methylation-resistant genes required the existence of active histone marks, whereas the loss of those marks strongly correlated with DNA methylation-sensitive genes.

## RESULTS

### Distribution of methylated and unmethylated CpG sites around the TSS

**Figure 1:**
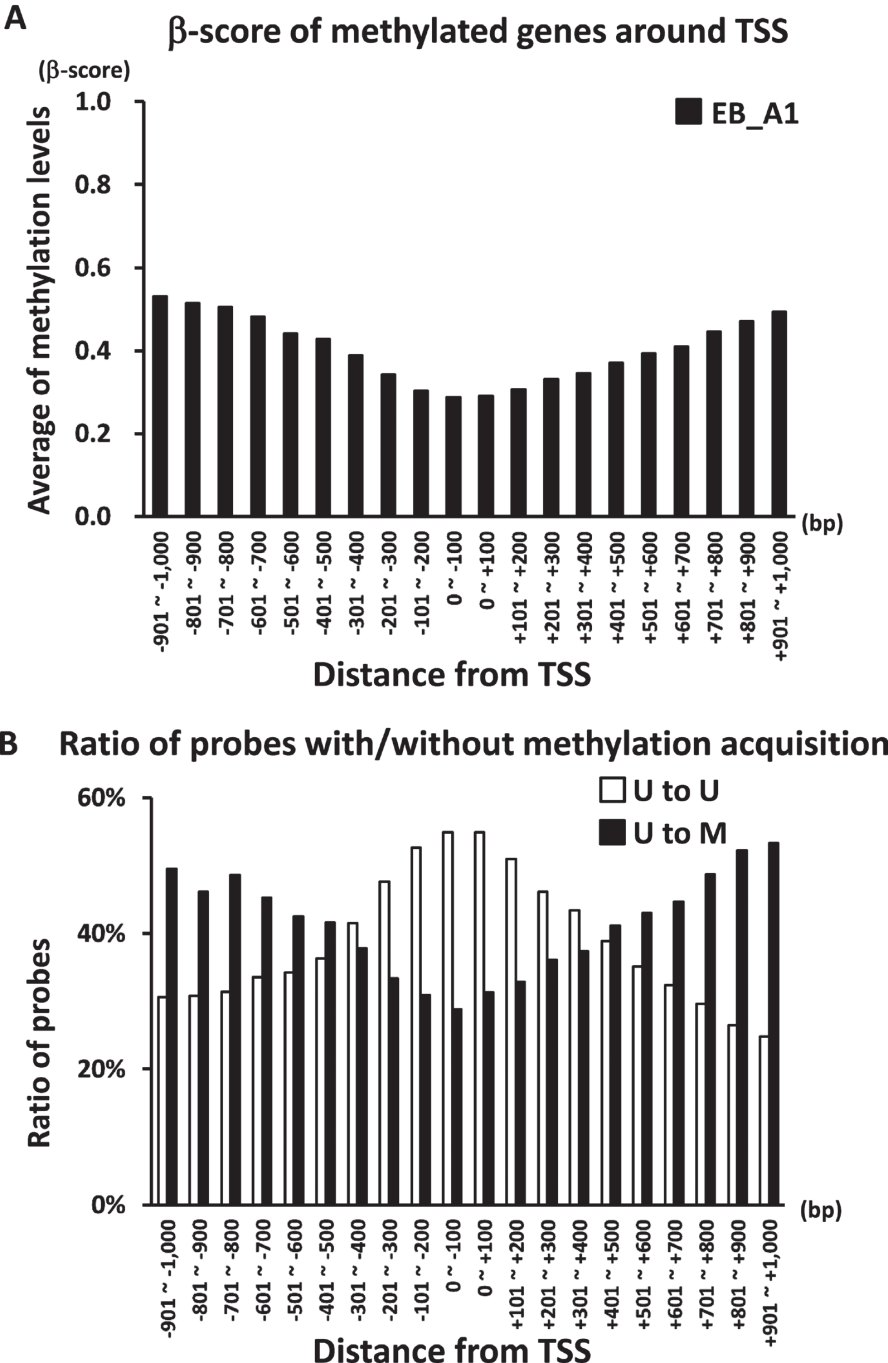
Distribution of DNA methylation status around the TSS after EBV infection (**A**) β-score of *de novo* methylated genes around the TSS. If the methylation status of an Infinium probe was altered from U in MKN7_WT to M in MKN7_EB_A1 cells, the probe was called “U to M” probe. If there were ≥ 1 “U to M” probes within ± 1,000 bp from the TSS, we regarded the genes as *de novo* methylation-induced genes, and 9,275 genes satisfied the criteria. The average of β-score of all the probes within ± 1,000 bp from the TSS of the 9,275 genes was calculated based on the distance from the TSS, and the distribution of DNA methylation levels around the TSS in MKN7_EB_A1 cells was shown. (**B**) Distribution of “U to U” and “U to M” probes around the TSS. Among the 9,275 methylation-induced genes, “U to U” and “U to M” probes were separately counted based on the distance from the TSS, and their ratios to all the probes are shown.

If the methylation status of an Infinium probe was altered from the unmethylated state (U; β-score ≤ 0.20) in MKN7_WT cells to a methylated state (M; β-score ≥ 0.40), the probe was called “U to M” probe. If there were ≥ 1 “U to M” probes within ± 1,000 bp from the TSS, we regarded the genes as *de novo* methylation-induced genes, and 9,275 genes satisfied the criteria. When we calculated the average of β-score of all the probes within ± 1,000 bp from the TSS of the 9,275 genes, based on the distance from the TSS, the elevated level of β-score was lower in the vicinity of the TSS (Figure 1A). Among the 9,275 methylation-induced genes, “U to U” and “U to M” probes were then separately counted, based on the distance from the TSS, and their ratios to all the probes were compared (Figure 1B). The ratio of “U to M” probes was lower in the vicinity of the TSS (within ± 400 bp from TSS), but that of “U to U” probes was inversely higher in the vicinity of the TSS. These findings suggested that unmethylated CpG sites were unevenly maintained or protected under DNA methylation induction during EBV infection.

### DNA methylation patterns during EBV infection

**Figure 2:**
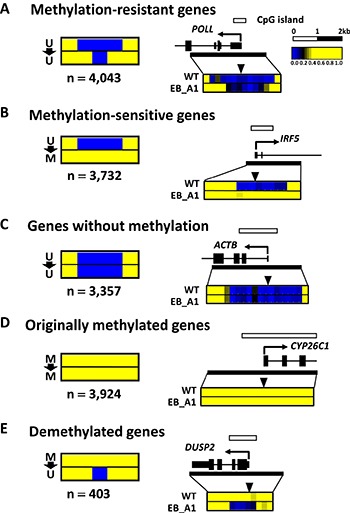
Patterns of DNA methylation acquisition during EBV infection (**A**) Methylation-resistant genes. Among the methylation-induced genes that had at least one “U to M” probe within ± 1000 bp from the TSS, 4,043 genes were found to have ≥ 2 consecutive “U to U” probes within ±500 bp from the TSS, and were defined as methylation-resistant genes. (**B**) Among the methylation-induced genes, 3,732 genes did not have consecutive “U to U” probes within ± 500 bp from the TSS, and were defined as methylation-sensitive genes. (**C**) In 3,357 genes without methylation, all the probes within ± 1,000 bp from the TSS were “U to U” probes. (**D**) In 3,924 originally methylated genes, all the probes within ± 1,000 bp from TSS were “M to M” probes. (**E**) In 403 demethylated genes, at least one probe was “M to U” probe within 500 bp from the TSS.

We further analyzed DNA methylation induction patterns during EBV infection. Among the methylation-induced genes, 4,043 genes were found to have ≥ 2 consecutive “U to U” probes within ± 500 bp from the TSS and defined as methylation-resistant genes (Figure 2A). In contrast, 3,732 genes did not have consecutive “U to U” probes within ± 500 bp from the TSS and defined as methylation-sensitive genes (Figure 2B). In 3,357 genes without methylation induction, all the probes within ± 1,000 bp from the TSS were “U to U” probes (Figure 2C). In 3,924 originally methylated genes, all the probes within ± 1,000 bp from the TSS were “M to M” probes (Figure 2D). A total of 403 genes had ≥ 1 “M to U” probes within ± 500 bp from the TSS and defined as demethylated genes (Figure 2E).

### Features of methylation-sensitive and resistant genes

**Figure 3:**
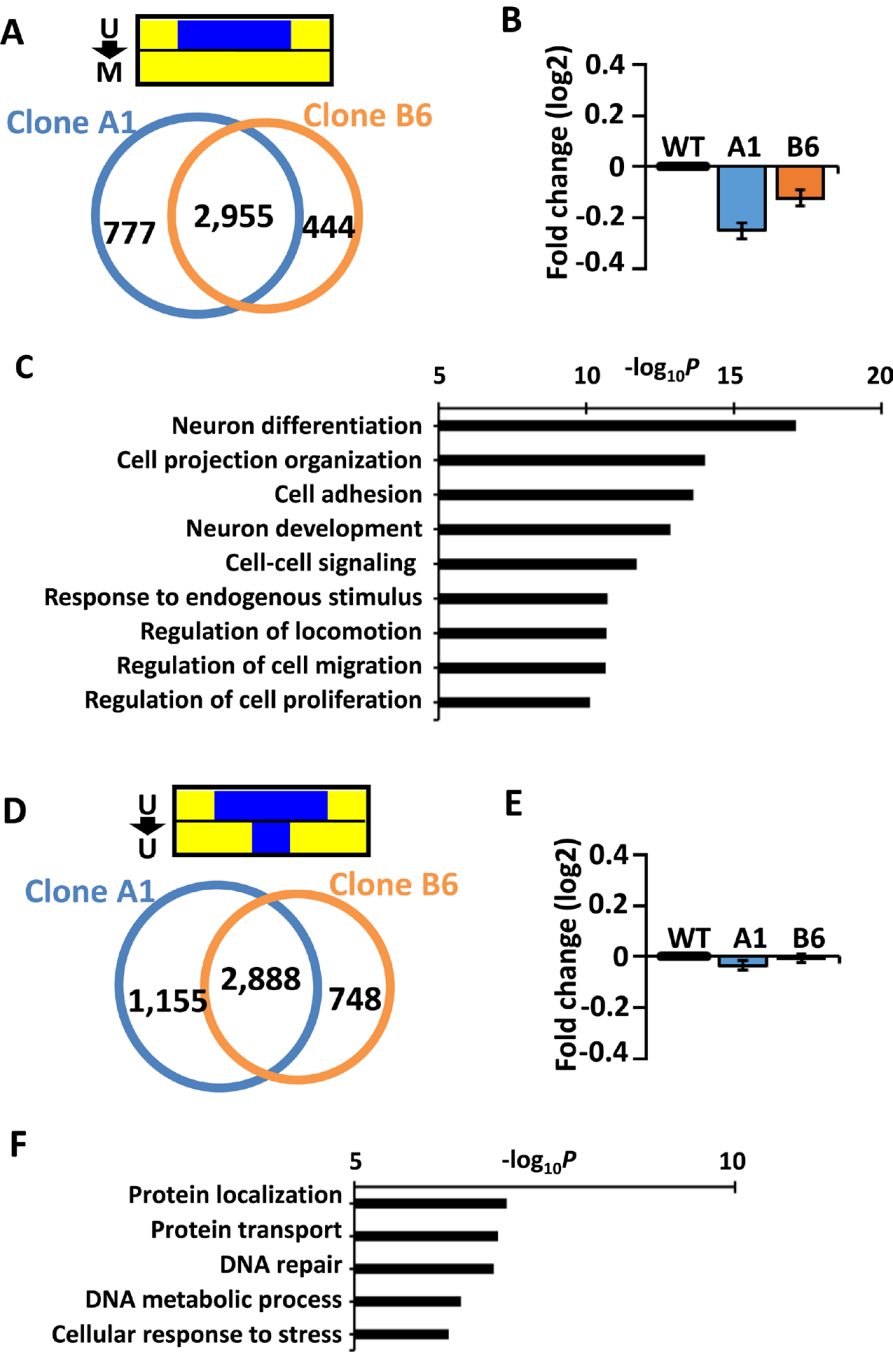
Characteristics of methylation-sensitive and methylation-resistant genes (**A**) The methylation-sensitive genes in MKN7_EB_A1 cells and those in MKN7_EB_B6 cells overlapped well. (**B**) The expression of methylation-sensitive genes in MKN7_WT, MKN7_EB_A1, and MKN7_EB_B6 cells was compared and is shown by fold expression change against WT. Methylation-sensitive genes were repressed in both MKN7_EB_A1 and MKN7_EB_B6 cells. (**C**) GO terms enriched in the overlapped methylation-sensitive genes were analyzed, and terms related to differentiation, cell adhesion, development, and regulation of cell proliferation were significant. (**D**) The methylation-resistant genes in MKN7_EB_A1 and MKN7_EB_B6 cells overlapped well. (**E**) The expression of methylation-resistant genes in WT, MKN7_EB_A1, and MKN7_EB_B6 cells was compared and is shown by fold expression change against WT. Expression levels of methylation-resistant genes in MKN7_WT cells were maintained in both MKN7_EB_A1 and MKN7_EB_B6 cells. (**F**) GO terms enriched in the overlapped methylation-resistant genes were analyzed, and terms related to DNA repair were significant.

The methylation-sensitive genes in the two independent EBV-infected MKN7 clones were compared and they overlapped well (Figure 3A). They were markedly repressed in both MKN7_EB_A1 and MKN7_EB_B6 clones (Figure 3B), and gene ontology (GO) terms significantly enriched in the overlapped methylation-sensitive genes were related to differentiation, cell adhesion, development, and regulation of cell proliferation, e.g., *CDH1*, *CDKN2A*, and *RHOB* (Figure 3C). Methylation acquisition in the whole promoter regions of genes related to differentiation and development as well as their repression are consistent with our previous finding that PRC target genes in ES cells were targets of aberrant methylation in EBV-infected gastric cancer cells [4].

The methylation-resistant genes in MKN7_EB_A1 and MKN7_EB_B6 clones were also well overlapped (Figure 3D). Their expression levels in MKN7_WT cells were retained in both EBV-infected clones (Figure 3E), and GO terms significantly enriched in the overlapped methylation-resistant genes were related to DNA repair, e.g., mismatch repair genes, including *MLH1*, *MSH2*, and *MSH6* (Figure 3F).

### Alteration of histone active marks

We next analyzed alteration of histone H3K4me3 and H3K27ac marks during EBV infection by ChIP-seq, and their correlations to gene expression and DNA methylation induction.

**Figure 4:**
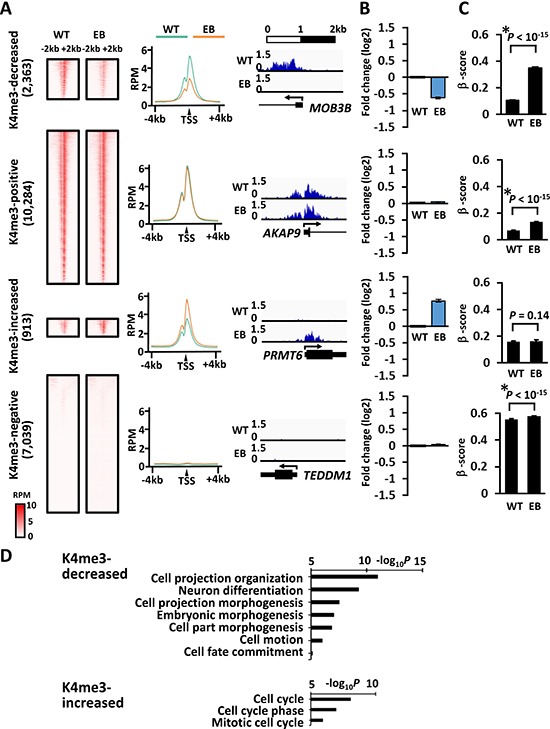
H3K4me3 alteration during EBV infection and its correlation to gene expression and DNA methylation induction (**A**) H3K4me3 signals at promoters in MKN7_WT and MKN7_EB_A1 cells. Compared with H3K4me3 signal around the TSS in MKN7_WT cells, that in MKN7_EB_A1 was decreased in 2,363 genes (*decreased*), remained positive in 10,284 genes (*positive*), increased in 913 genes (*increased*), and remained negative in 7,039 genes (*negative*). The heatmap shows the distribution of H3K4me3 signals of all the genes included in each subset within ± 2 kb from the TSS (*left*). The average profile plot shows the distribution of averaged H3K4me3 signal within ± 4 kb from the TSS (*middle*). Distribution of ChIP-seq reads of a representative gene is shown (*right*). (**B**) The gene expression was compared between MKN7_WT and MKN7_EB_A1 cells, and presented as fold expression change against WT. Gene expression was markedly downregulated in “decreased” genes (*top*), retained in “positive” genes (*second*), markedly upregulated in “increased” genes (*third*), and retained in “negative” genes (*bottom*). (**C**) As for DNA methylation alteration, β-score of each gene at the probe nearest to the TSS was compared between MKN7_WT and MKN7_EB_A1 cells, and presented as the average and SE. The methylation level was markedly elevated in “decreased” genes (*top*), retained at unmethylated state in “positive” (*second*) and “increased” genes (*third*), and retained at methylated state in “negative” genes (*bottom*). (**D**) GO term enrichment in genes with increased and decreased H3K4me3 signal. Genes related to differentiation and morphogenesis were significantly enriched in genes with decreased H3K4me3 signal, whereas genes related to cell cycle were significantly enriched in genes with increased H3K4me3 signal.

H3K4me3 signal around the TSS in MKN7_EB_A1 cells was decreased in 2,363 genes, remained positive in 10,284 genes, remained negative in 7,039 genes, and increased in 913 genes compared to that in MKN7_WT (Figure 4A). Compared to that in MKN7_WT cells, gene expression was markedly downregulated in genes with decreased H3K4me3, but markedly upregulated in genes with increased H3K4me3 (Figure 4B). Consistently, the DNA methylation level was markedly elevated from 0.11 ± 0.00 to 0.35 ± 0.01 (average β-score ± SE) in genes with decreased H3K4me3, which significantly included differentiation- and morphogenesis- related genes (Figure 4C, 4D). However, DNA methylation level was retained at unmethylated state from 0.16 ± 0.01 to 0.16 ± 0.01 (β-score) in genes with increased H3K4me3, which significantly included cell cycle related genes.

**Figure 5:**
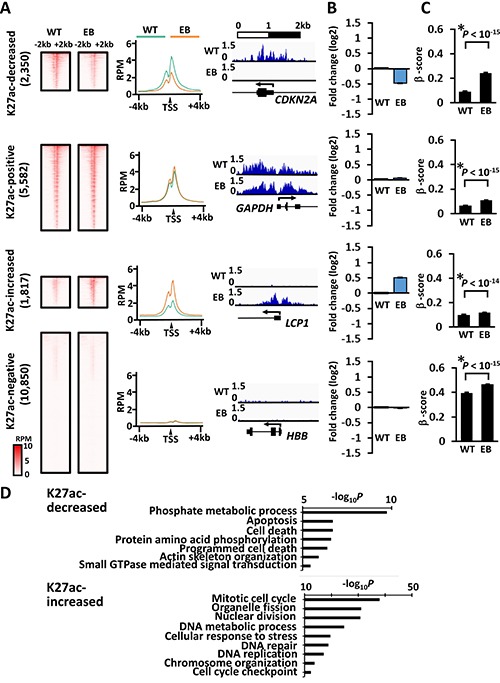
H3K27ac alteration during EBV infection, and its correlation to gene expression and DNA methylation induction (**A**) H3K27ac signals at promoters in MKKN7_WT and MKN7_EB_A1 cells. Compared with H3K27ac signal around the TSS in MKN7_WT cells, that in MKN7_EB_A1 cells was decreased in 2,350 genes (*decreased*), remained positive in 5,582 genes (*positive*), increased in 1,817 genes (*increased*), and remained negative in 10,850 genes (*negative*). The heatmap shows the distribution of H3K27ac signals of all the genes included in each subset within ± 2 kb from the TSS (*left*). The average profile plot shows the distribution of averaged H3K27ac signal within ± 4 kb from the TSS (*middle*). Distribution of ChIP-seq reads of a representative gene is shown (*right*). (**B**) The gene expression was compared between MKN7_WT and MKN7_EB_A1 cells and presented as fold expression change against WT. Gene expression was markedly downregulated in “decreased” genes (*top*), retained in “positive” genes (*second*), markedly upregulated in “increased” genes (*third*), and retained in “negative” genes (*bottom*). (**C**) As for DNA methylation alteration, β-score of each gene at the probe nearest to the TSS was compared between MKN7_WT and MKN7_EB_A1 cells, and presented as the average and SE. The methylation level was markedly elevated in “decreased” genes (*top*), retained at unmethylated state in “positive” (*second*) and “increased” genes (*third*), and retained at high levels in “negative” genes (*bottom*). (**D**) GO term enrichment in genes with increased and decreased H3K27ac signal. Genes related to cell cycle and DNA repair were significantly enriched in genes with increased H3K27ac signal, whereas genes related to apoptosis and cell death were significantly enriched in genes with decreased H3K27ac signal.

H3K27ac signals at promoters were similarly analyzed in MKN7_WT and MKN7_EB_A1 cells. H3K27ac signal around the TSS in MKN7_EB_A1 cells was decreased in 2,350 genes, remained positive in 5,582 genes, remained negative in 10,850 genes, and increased in 1,817 genes compared to that in MKN7_WT cells (Figure 5A). Compared to that in MKN7_WT cells, the gene expression was markedly downregulated in genes with decreased H3K27ac signal, but markedly upregulated in genes with increased H3K27ac signal (Figure 5B). Similar to H3K4me3, DNA methylation level was markedly elevated from 0.09 ± 0.00 to 0.24 ± 0.01 (β-score) in genes with decreased H3K27ac, but retained at unmethylated state from 0.10 ± 0.00 to 0.12 ± 0.00 (β-score) in genes with increased H3K27ac (Figure 5C). Significantly enriched GO terms were related to apoptosis and cell death in genes with decreased H3K27ac signal (Figure 5D); repression and DNA methylation of these genes were suggested to contribute to tumorigenesis. Significantly enriched GO terms in increased H3K27ac signal were related to cell cycle and DNA repair; retained expression of these genes was also suggested to contribute to tumorigenesis.

To confirm that these histone modification and gene expression alteration were not due to long-term passage of cells but due to EBV infection, we also analyzed MKN7 cells cultured for the same period (Supplementary Figure 1). Decrease or increase of H3K4me3 and H3K27ac, and the subsequent gene expression alterations, were not generally observed during the long-term passage.

### Alteration of histone repressive mark

We next analyzed alteration of histone H3K27me3 mark during EBV infection by ChIP-seq and its correlation to gene expression and DNA methylation induction.

**Figure 6:**
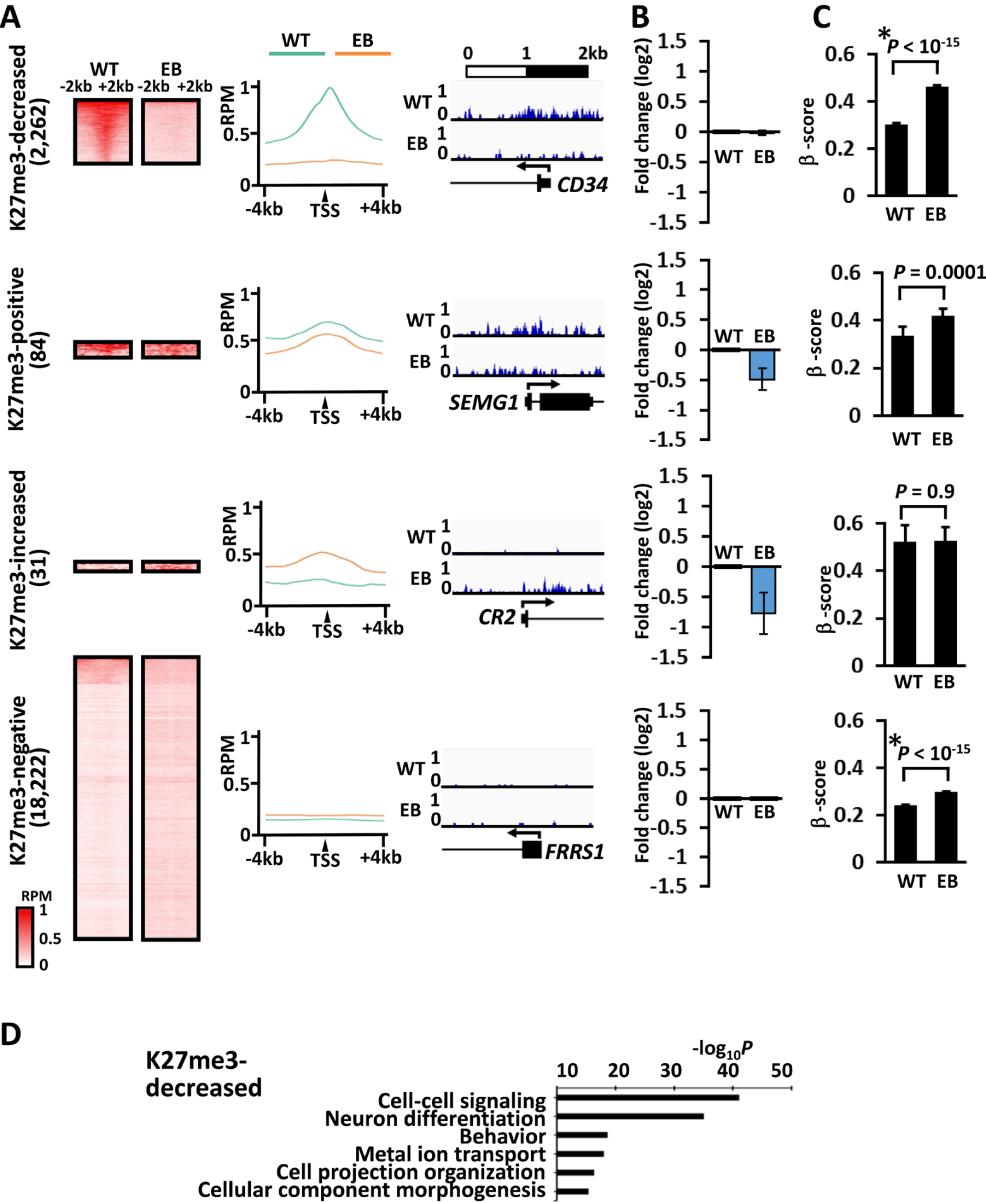
H3K27me3 alteration during EBV infection and its correlation to gene expression and DNA methylation induction (**A**) H3K27me3 signals at promoters in MKKN7_WT and MKN7_EB_A1 cells. Compared with H3K27me3 signal around the TSS in MKN7_WT cells, that in MKN7_EB_A1 cells was decreased in 2,262 genes (*decreased*), remained positive in as few as 84 genes (*positive*), increased in as few as 31 genes (*increased*), and remained negative in 18,222 genes (*negative*). The heatmap shows the distribution of H3K27me3 signals of all the genes included in each subset within ± 2 kb from the TSS (*left*). The average profile plot shows the distribution of averaged H3K27me3 signal within ± 4 kb from the TSS (*middle*). Distribution of ChIP-seq reads of a representative gene is shown (*right*). (**B**) The gene expression was compared between MKN7_WT and MKN7_EB_A1 cells and presented as fold expression change against WT. Gene expression was not upregulated in “decreased” genes despite of loss of repressive H3K27me3 histone mark (*top*) and was retained in “negative” genes (*bottom*). (**C**) As for DNA methylation alteration, β-score of each gene at the probe nearest to the TSS was compared between MKN7_WT and MKN7_EB_A1 cells and presented as the average and SE. The methylation level was markedly elevated to methylated state in “decreased” genes during the loss of repressive H3K27me3 histone mark (*top*). (**D**) GO term enrichment in genes with decreased H3K27me3 signal. Genes related to differentiation and morphogenesis were significantly enriched in genes with increased H3K27me3 signal.

H3K27me3 signal around the TSS in MKN7_EB_A1 cells was decreased in 2,262 genes, and remained negative in 18,222 genes compared to that in MKN7_WT cells (Figure 6A). Genes maintaining positive H3K27me3 signal or showing increased H3K27me3 signal were rarely observed. Compared to that in MKN7_WT cells, gene expression was not upregulated in genes with decreased H3K27me3 mark, but low expression levels were maintained in MKN7_EB_A1. Gene expression was also retained in genes retaining negative H3K27me3 signal (Figure 6B). DNA methylation level was markedly elevated from 0.30 to 0.46 (β-score) in genes with decreased H3K27me3, suggesting a switch of repressive epigenetic modifications from H3K27me3 to DNA methylation.

### Integrated analysis of all the histone marks and DNA methylation

**Figure 7:**
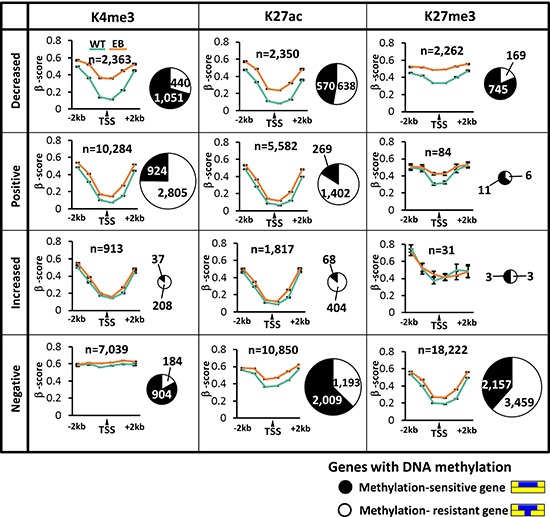
Ratio of methylation-sensitive and resistant genes and its association with histone modification alteration patterns As for each of 12 histone modification alteration patterns (See Figures 4, 5, and 6), DNA methylation levels were assessed by calculating the average of β-scores based on the distance from the TSS. The Pie chart shows the number of the methylation-sensitive genes (*black*) and methylation-resistant genes (*white*).

As for each histone modification alteration pattern (Figures 4–6), DNA methylation levels in MKN7_WT and MKN7_EB_A1 cells were assessed by calculating the average of β-scores, based on the distance from the TSS (Figure 7). When signals of histone active marks were decreased or retained at lower levels, i.e., were low after EBV infection, these genes were significantly associated with methylation-sensitive genes showing elevated DNA methylation level in whole promoter regions, including the vicinity of the TSS. When signals of histone active marks were increased or retained at high levels, i.e., were high after EBV infection, these genes were significantly associated with methylation-resistant genes, showing protection of unmethylated state in the vicinity of the TSS. Genes losing H3K27me3 were significantly associated with DNA methylation-sensitive genes, as mentioned above.

**Figure 8:**
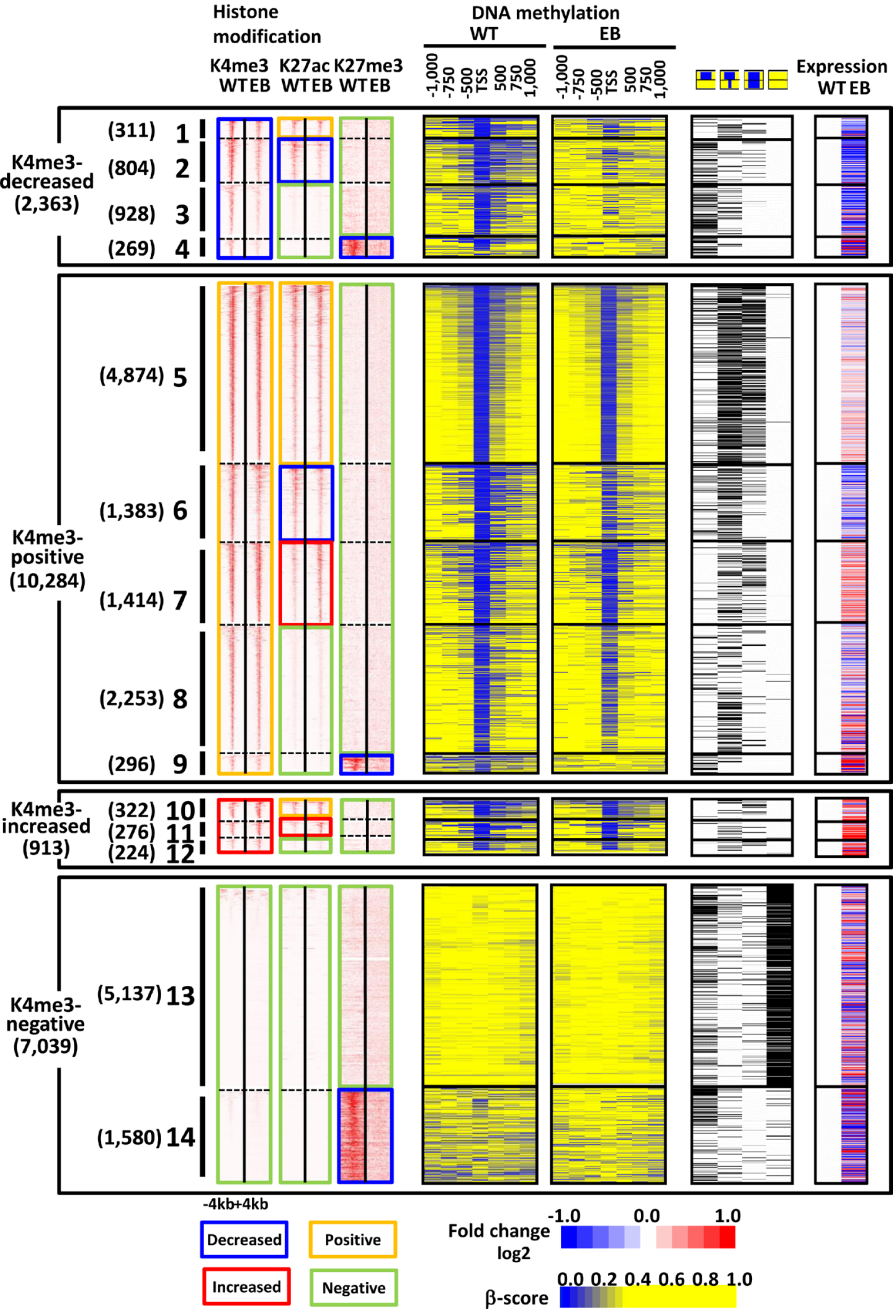
Integrated analysis of all the histone marks and DNA methylation Genes decreasing H3K4me3 signal generally related to DNA methylation-sensitive genes and gene repression, but were further classified into four subgroups (*#1-#4*). Whereas genes also losing H3K27ac (*#2*) and retaining H3K27ac-negative status (*#3*) strongly associated with DNA methylation-sensitive genes and gene repression, genes retaining H3K27ac-positive status (*#1*) rather associated with DNA methylation-resistant genes and were thus less downregulated. Genes retaining H3K4me3-positive status generally related to DNA methylation-resistant genes, but were further classified into five subgroups (*#5-#9*). Whereas genes without H3K27me3 mark (*#5-#8*) strongly associated with DNA methylation-resistant genes regardless H3K27ac status, genes losing H3K27me3 mark (*#9*) strongly associated with DNA methylation-sensitive genes. Gene expression alteration in subgroups *#5-#8* depended on H3K27ac alteration patterns. Genes with increased H3K4me3 signal generally related to DNA methylation-resistant genes (*#10-#12*). Genes retaining H3K4me3-negative status were further classified into two subgroups (*#13-#14*). Whereas genes retaining H3K27ac-negative and H3K27me3-negative status (*#13*) strongly associated with originally methylated genes, genes losing H3K27me3 mark associated with DNA methylation-sensitive genes. Regardless of H3K4me3 status, genes losing H3K27me3 mark at promoters (*#4, #9, #14*) associated with DNA methylation-sensitive genes.

If the decrease in H3K4me3 occurred in H3K27ac(+) genes, the genes likely showed decrease of H3K27ac (Figure 8, subgroup 2). If decrease in H3K4me3 occurred in H3K27ac(−) genes, the genes maintained H3K27ac(−) status and hardly gained H3K27ac (Figure 8, subgroups 3–4). Decrease in H3K4me3 thus hardly coexists with high level of H3K27ac after EBV infection. Whereas the decreases of these active marks were correlated with *de novo* DNA methylation, the genes losing H3K4me3, but keeping H3K27ac(+) status were significantly correlated with methylation-resistant genes, implying that not only H3K4me3, but also modulators for H3K27ac might also work as resistant factors for DNA methylation induction (Figure 8).

Genes maintaining H3K4me3(+) status with decreased H3K27ac (Figure 8, subgroup 6) or maintained H3K27ac(−) status (Figure 8, subgroups 8–9) were mostly DNA methylation-resistant genes. However, genes maintaining both H3K4me3(+) and H3K27ac(+) status, abundantly included DNA methylation-resistant genes as well as unmethylated genes (Figure 8, subgroup 5). The unmethylated genes are mostly H3K4me3-positive genes, whose H3K27ac status is mostly retained positive (Figure 8, subgroup 5) or increased (Figure 8, subgroup 7).

Genes with increased H3K4me3 signal generally related to DNA methylation-resistant genes (Figure 8, subgroups 10–12).

Among genes maintaining H3K4me3(−) status, those with H3K27me3 pre-mark likely lost H3K27me3, but acquire *de novo* DNA methylation instead (Figure 8, subgroup 14). Those without H3K27me3 pre-mark markedly correlated with already methylated genes; these genes were generally negative for all three histone modifications (Figure 8, subgroup 13).

## DISCUSSION

We previously reported that EBV-positive gastric cancer is distinguished by extensive DNA hypermethylation of gene promoters, and EBV itself is the cause of *de novo* DNA methylation [4]. Some genes, named common marker genes, are commonly methylated in low-methylation, high-methylation/MSI+, and extensively high-methylation/EBV+ gastric cancer cases; MKN7, a low-methylation gastric cancer cell line, shows methylation in these genes, while GES1, a normal gastric epithelial cell immortalized with SV40, is not methylated in these genes. EBV infection was shown to cause genome-wide methylation and thus induce EBV-CIMP in both MKN7 and GES1 [4, 7]. Here, we show that DNA methylation-induced genes in EBV-infected MKN7 were generally classified into “DNA methylation-sensitive” and “DNA methylation-resistant” depending on methylation status around the TSS, that histone modification was also dynamically altered with EBV infection, and that histone modification alteration occurred in a coordinated manner with DNA methylation alteration.

Among the *de novo* methylated promoter regions, roughly half acquired complete methylation in the whole promoter region (Figure 2A), which led to repression of the downstream genes (Figure 3B). These DNA methylation-sensitive genes included known anti-oncogenic genes acting in cell adhesion or apoptosis (Figure 3C). The inactivation of *CDH1* was observed with TSS methylation, and this may contribute to the characteristic feature of EBV-positive gastric cancer that they often appear as diffuse-type gastric cancer [16, 17]. *CDKN2A* was also a DNA methylation-sensitive gene, being consistent with previous reports that *CDKN2A* is hypermethylated and silenced in EBV-positive gastric cancer [6].

In contrast, the other half acquired *de novo* methylation in the surrounding region of the promoters, but protected unmethylated status at the immediate vicinity of the TSS (Figure 2B). These genes maintained gene expression (Figure 3E), and were regarded as DNA methylation-resistant genes. These were significantly enriched with DNA repair genes (Figure 3F), e.g., mismatch repair genes, including *MLH1*, *MSH2*, and *MSH6*. This supports the previous report that EBV-positive gastric cancer does not show *MLH1* methylation and is thus microsatellite stable [5, 18]. The existence of DNA methylation-resistant genes and its reproducibility among EBV-infected clones suggest that there might be some mechanisms protecting TSS from *de novo* DNA methylation pressure of EBV infection [19].

Dynamic alteration was detected in both active and repressive histone marks. Approximately 2,000 to 4,000 genes showed alteration of H3K4me3, H3K27ac, and H3K27me3 levels at promoter regions. The activated genes with increase of H3K27ac were significantly enriched with DNA repair genes (Figure 5D). As similar GO terms were enriched in DNA methylation-resistant genes, genes with gain of H3K27ac significantly correlated with DNA methylation-resistant genes (Figure 7). *BIRC5*, which negatively regulates apoptosis and was reportedly upregulated by EBV latent protein LMP2 [20], showed increased H3K27ac signal with upregulated expression. In contrast, decrease in H3K27ac was observed in 2,350 genes (Figure 5A), which significantly included genes related to apoptosis and cell death (Figure 5D). These genes were significantly repressed, and strongly correlated with DNA methylation-sensitive genes (Figure 7). However, 161 among the 2,350 genes did not acquire *de novo* DNA methylation, and were downregulated during EBV infection. For example, genes related to significantly enriched GO terms of cell adhesion and apoptosis, included *COL4A3* [21, 22], *PHLPP1* [23], and *TIA1* [24], which were reportedly downregulated in cancer. These genes did not acquire *de novo* DNA methylation, but showed decrease of H3K27ac and downregulation of gene expression. It was suggested that histone modification alteration induced by EBV infection might regulate critical tumor-related genes without necessarily accompanying DNA methylation alteration. While EBV reportedly established its latency by changing histone modifications of viral genome as well as DNA methylation of viral genome [25, 26], EBV might also alter histone modification of host genome and contribute to tumorigenesis.

Genes with decrease of H3K4me3 or H3K27me3 signals significantly included differentiation-related genes (Figure 4D and 6D) and were accompanied with *de novo* DNA methylation with gene repression (Figures 4B and 6B). In contrast, the methylation-resistant genes and the unmethylated genes were marked with both or one of active histone marks (Figure 8). It is reported that active promoters with H3K4me3 are protected from *de novo* methylation by occupying transcription factors and histone methyltransferases at promoter regions. *De novo* methyltransferases, *DNMT3A, 3B* and *3L*, are known to have ADD domain that recognize H3K4me0 [27, 28]. The polycomb repressive complex, for example, establishes and maintains H3K27me3 marks, which were suggested to be pre-marks for *de novo* DNA hypermethylation [29–32]. The switch of epigenetic repression marks from H3K27me3 to DNA methylation was observed in H3K4me3(+) genes as well as H3K4me3(−) genes in this study, suggesting that H3K27me3 pre-marks might be strong predisposed promoters rather than H3K4me0 status that recruits DNA methyltransferases [27–32].

While DNA methylation induction was found to be preferentially accompanied with loss of histone active marks or repressive mark, it is yet to be investigated which epigenetic alteration occurs first and could causally induce other alterations. The efficiency of *in vitro* EBV infection into gastric cells is as low as < 0.001% [7], so that time series analysis of histone modification alteration is difficult at early time points. Improvement of infection efficiency and ChIP technique using smaller number of cells is now underway [Okabe et al. unpublished data], which will enable us elucidate the order of epigenetic alterations in host genome, as well as viral genome [7]. The analysis using non-neoplastic gastric epithelial cell GES1 should also help us understand the whole mark of epigenomic alteration in gastric epithelial cell induced by EBV infection.

In conclusion, histone modification altered coordinately with DNA methylation alteration during EBV infection. H3K27me3 pre-marks and decrease of active histone marks were strongly correlated with DNA methylation-sensitive genes.

## MATERIALS AND METHODS

### Cells

We obtained MKN7, a low DNA methylation phenotype gastric cancer cell line, from Riken BioResource Center (Ibaraki, Japan). We infected recombinant EBV into MKN7 cells (MKN7_WT) using the Akata system [33] to establish two independent EBV-infected MKN7 clones (MKN7_EB_A1 and MKN7_EB_B6) in our previous study [4]. MKN7_WT and EBV-infected MKN7 clones were cultured at 37°C in a 5% CO2 incubator in RPMI 1640 medium (Wako, Tokyo, Japan) with 10% fetal bovine serum (HyClone SH30910.03; GE Healthcare, Chicago, IL, USA), streptomycin sulfate (100 mg/L) (Sigma-Aldrich, St. Louis, MO, USA), and penicillin G sodium (100 mg/L) (Sigma-Aldrich). EBV-infected MKN7 clones were selected by 300 g/mL G418 (Life technologies, Carlsbad, CA, USA). These clones were cultured for 18 weeks after EBV infection for selection and cloning. EBER *in situ* hybridization was performed, as previously described [4], to confirm existence of EBV in each cancer cell (Supplementary Figure 2). These cells underwent the subsequent analyses of DNA methylation and histone modification. MKN7 cells cultured for the same period of 18 weeks, named MKN7_EB(−) cells, also underwent histone modification analyses, while we previously reported that MKN7_EB(−) cells did not acquire aberrant DNA methylation or gene silencing [4]. MKN7 cells were transfected with a vacant vector pcDNA3.1 (Invitrogen, Carlsbad, CA, USA) with neomycin resistant gene, and it was also already confirmed that long exposure to neomycin for selection did not cause aberrant DNA methylation or gene silencing [7].

### Infinium assays

High-resolution methylation analysis was conducted on the Illumina Infinium HumanMethylation450 BeadChip array. Illumina Infinium HumanMethylation450 BeadChip array contains over 485,000 CpG sites and covers 99% of RefSeq genes. There were 177,656 CpG sites within 1,000 bp (from − 500 bp to + 500 bp) around the TSS of 25,593 RefSeq genes on autosomal chromosomes, with an average of 6.8 CpG sites and a median of 7.0 CpG sites covered in Infinium 450K.

Five hundred nanograms of DNA from cultured cells were extracted by using QIAamp DNA Mini Kit (Qiagen, Hilden, Germany) and underwent bisulfite conversion using EZ DNA Methylation Kit (Zymo Research, Irvine, CA, USA) according to the manufacturer's protocol. Bisulfite-converted DNA was subsequently amplified, fragmented, and hybridized to the BeadChip. After single base extension and staining, the BeadChip was scanned by using the Illumina HiScan SQ scanner following the manufacturer's instructions. Infinium data were submitted to the GEO DataSets, and the accession number is GSE89269.

For each CpG site, the methylation rate was calculated as the value ranging from 0.00 (completely unmethylated) to 1.00 (completely methylated), based on the fluorescence signal intensity of the probe. When β-score was ≤ 0.20, the CpG site was considered unmethylated (U) and the probe was defined as a “U” probe. When β-score was ≥ 0.40, the CpG site was considered methylated (M), and the probe was defined as an “M” probe. When the methylation status was altered from U to M, or from M to U, the probe was called a “U to M” probe or a “M to U” probe, respectively. When the U probe still showed unmethylated state after EBV infection, the probe was called a “U to U” probe. Similarly, when the M probe still showed methylated state after EBV infection, the probe was called “M to M” probe.

### Expression array analysis

Gene expression in MKN7_WT, MKN7_EB_A1, and MKN7_EB_B6 cells was analyzed by Affymetrix GeneChip Human Genome U133 plus 2.0 oligonucleotide arrays (Affimetrix, Fremont, CA, USA) as previously described [4]. Data were collected and analyzed by GeneChip Scanner 3000 (Affymetrix). The GeneChip data were analyzed using the Affymetrix GeneChip Operating Software v1.3 by MAS5 algorithms, to obtain signal value (GeneChip score) for each probe set. For global normalization, the average signal in an array was made equal to 100. Expression array data were submitted to the GEO DataSets, and the accession number is GSE31789.

### GO analysis

Gene annotation enrichment analysis was performed using the Functional Annotation tool at DAVID 6.7 Bioinformatics Resources (https://david.ncifcrf.gov/home.jsp). The GO terms were filtered to select terms from the biological process (BP) subontology.

### Chromatin immunoprecipitation (ChIP)

ChIP was performed as previously described [34]. MKN7_WT and MKN7_EB_A1 cells were cross-linked with 1% formaldehyde for 10 min, followed by quenching with 125 mM glycine for 5 min. DNA fragmentation was performed by using UD 201 (Tomy, Tokyo, Japan) with the following settings: output level 2, 50% duty, 15 sec, 5 cycles, on floating ice to obtain 200–500 bp DNA fragments. An aliquot of chromatin was removed as input and the remainder of the chromatin was used for ChIP analysis using anti-H3K4me3 (ab8580, Abcam, Cambridge, MA, USA, 2 μL per ChIP), anti-H3K27ac (CMA309, MBL, Nagoya, Japan, 2 μL per ChIP), and anti-H3K27me3 (07–142, Millipore, Darmstadt, Germany, 2 μL per ChIP) antibodies.

The fragmented samples were incubated with antibodies bound to protein A-sepharose beads (GE Healthcare) or Dynabeads (Life technologies) at 4°C overnight. The beads were then washed eight times and eluted with elution buffer. The eluates and input were treated with pronase at 42°C for 2 h and then underwent incubation at 65°C overnight to reverse the cross-linking. The immunoprecipitated and input DNA were purified by 2 extractions with phenol:chloroform, followed by ethanol precipitation. Purified DNA samples were resuspended in 20 μL of Tris-HCL (pH 8.0). The concentration of the DNA sample was measured with a Qubit fluorometer (Life Technologies).

### ChIP-seq analysis

The ChIP DNA was used to prepare library samples following the manufacturer's instructions (Illumina). Deep sequencing was performed on the Solexa Genome Analyzer II to obtain 36-bp single reads. Sequencing tags were mapped to UCSC build hg19 (GRCh37) assembly of the human genome using ELAND. Window sizes of 300 bp for H3K4me3 and H3K27ac and 500 bp for H3K27me3 were used to calculate the number of mapped reads per million reads (RPM) via the Galaxy workflow system [35]. The highest RPM within ± 1,000 bp around the TSS was regarded as the represented RPM.

Then, represented RPM of H3K4me3 and H3K27ac were normalized by per-sample scale factors based on the previous report [36]. Briefly, a set of genes were selected which expressed high level (array score > 200) and had low variance (variance in −0.1 < log2 fold change < 0.1) in both MKN7 and MKN7_EB_A1 cells. A total of 1,023 genes meet the criteria and the average of represented RPM of those genes was considered to reflect the variability in read count due to technical differences in sample preparation. The normalized RPM were visualized using IGV browser and ngs.plot [37].

A histone mark was recognized as increased when the number of represented RPM of MKN7_WT cells was greater than the cut-off RPM and MKN7_EB_A1/MKN7_WT was > 1.5. A histone mark was recognized as lost when the number of represented RPM of MKN7_EB_A1 cells was greater than the cut-off RPM and MKN7_EB_A1/MKN7_WT <2/3. The cut-off RPM was 2.0 for H3K4me3 and H3K27ac, and 1.0 for H3K27me3.

### Statistical analysis

Gene expression levels by GeneChip score and methylation levels by β-score are presented as mean ± SE. Student's *t*-test was conducted for the comparison of two groups. Correlation between DNA methylation status and histone modification status was analyzed by χ^2^ test. R program (www.r-project.org/) was used in these analyses.

## SUPPLEMENTARY MATERIALS FIGURES



## Abbreviations

EBV: Epstein-Barr virus
WT: wild type
M: methylated
U: unmethylated
TSS: transcription start site
ChIP: Chromatin immunoprecipitation
RPM: number of mapped reads per million reads
GO: gene ontology.

## References

[1] Feinberg AP, Ohlsson R, Henikoff S (2006). The epigenetic progenitor origin of human cancer. Nat Rev Genet.

[2] Jones PA (2012). Functions of DNA methylation: islands, start sites, gene bodies and beyond. Nat Rev Genet.

[3] Funata S, Fukayama M, Kaneda A (2016). De NovoMethylation in. Cancer.

[4] Matsusaka K, Kaneda A, Nagae G, Ushiku T, Kikuchi Y, Hino R, Uozaki H, Seto Y, Takada K, Aburatani H, Fukayama M (2011). Classification of Epstein-Barr virus-positive gastric cancers by definition of DNA methylation epigenotypes. Cancer Res.

[5] Wang K, Yuen ST, Xu J, Lee SP, Yan HH, Shi ST, Siu HC, Deng S, Chu KM, Law S, Chan KH, Chan AS, Tsui WY (2014). Whole-genome sequencing and comprehensive molecular profiling identify new driver mutations in gastric cancer. Nat Genet.

[6] Cancer Genome Atlas Research Network (2014). Comprehensive molecular characterization of gastric adenocarcinoma. Nature.

[7] Matsusaka K, Funata S, Fukuyo M, Seto Y, Aburatani H, Fukayama M, Kaneda A (2017). Epstein-Barr virus infection induces genome-wide de novo DNA methylation in non-neoplastic gastric epithelial cells. J Pathol.

[8] Young LS, Rickinson AB (2004). Epstein-Barr virus: 40 years on. Nat Rev Cancer.

[9] Takada K (2000). Epstein-Barr virus and gastric carcinoma. Mol Pathol.

[10] Barski A, Cuddapah S, Cui K, Roh TY, Schones DE, Wang Z, Wei G, Chepelev I, Zhao K (2007). High-resolution profiling of histone methylations in the human genome. Cell.

[11] Chi P, Allis CD, Wang GG (2010). Covalent histone modifications--miswritten, misinterpreted and mis-erased in human cancers. Nat Rev Cancer.

[12] Hattori N, Ushijima T (2014). Compendium of aberrant DNA methylation and histone modifications in cancer. Biochem Biophys Res Commun.

[13] Ma J, Wang JD, Zhang WJ, Zou B, Chen WJ, Lam CS, Chen MH, Pang R, Tan VP, Hung IF, Lan HY, Wang QY, Wong BC (2010). Promoter hypermethylation and histone hypoacetylation contribute to pancreatic-duodenal homeobox 1 silencing in gastric cancer. Carcinogenesis.

[14] Muratani M, Deng N, Ooi WF, Lin SJ, Xing M, Xu C, Qamra A, Tay ST, Malik S, Wu J, Lee MH, Zhang S, Tan LL (2014). Nanoscale chromatin profiling of gastric adenocarcinoma reveals cancer-associated cryptic promoters and somatically acquired regulatory elements. Nat Commun.

[15] Ooi WF, Xing M, Xu C, Yao X, Ramlee MK, Lim MC, Cao F, Lim K, Babu D, Poon LF, Lin Suling J, Qamra A, Irwanto A (2016). Epigenomic profiling of primary gastric adenocarcinoma reveals super-enhancer heterogeneity. Nat Commun.

[16] Sudo M, Chong JM, Sakuma K, Ushiku T, Uozaki H, Nagai H, Funata N, Matsumoto Y, Fukayama M (2004). Promoter hypermethylation of E-cadherin and its abnormal expression in Epstein-Barr virus-associated gastric carcinoma. Int J Cancer.

[17] Yamamoto E, Suzuki H, Takamaru H, Yamamoto H, Toyota M, Shinomura Y (2011). Role of DNA methylation in the development of diffuse-type gastric cancer. Digestion.

[18] Kang GH, Lee S, Kim WH, Lee HW, Kim JC, Rhyu MG, Ro JY (2002). Epstein-Barr Virus-Positive Gastric Carcinoma Demonstrates Frequent Aberrant Methylation of Multiple Genes and Constitutes CpG Island Methylator Phenotype-Positive Gastric Carcinoma. The American Journal of Pathology.

[19] Namba-Fukuyo H, Funata S, Matsusaka K, Fukuyo M, Rahmutulla B, Mano Y, Fukayama M, Aburatani H, Kaneda A (2016). TET2 functions as a resistance factor against DNA methylation acquisition during Epstein-Barr virus infection. Oncotarget.

[20] Hino R, Uozaki H, Inoue Y, Shintani Y, Ushiku T, Sakatani T, Takada K, Fukayama M (2008). Survival advantage of EBV-associated gastric carcinoma: survivin up-regulation by viral latent membrane protein 2A. Cancer Res.

[21] Ando H, Aihara R, Ohno T, Ogata K, Mochiki E, Kuwano H (2009). Prognostic significance of the expression of MUC1 and collagen type IV in advanced gastric carcinoma. Br J Surg.

[22] Nie XC, Wang JP, Zhu W, Xu XY, Xing YN, Yu M, Liu YP, Takano Y, Zheng HC (2013). COL4A3 expression correlates with pathogenesis, pathologic behaviors, and prognosis of gastric carcinomas. Hum Pathol.

[23] Liu J, Weiss HL, Rychahou P, Jackson LN, Evers BM, Gao T (2009). Loss of PHLPP expression in colon cancer: role in proliferation and tumorigenesis. Oncogene.

[24] Reyes R, Alcalde J, Izquierdo JM (2009). Depletion of T-cell intracellular antigen proteins promotes cell proliferation. Genome Biol.

[25] Arvey A, Tempera I, Tsai K, Chen HS, Tikhmyanova N, Klichinsky M, Leslie C, Lieberman PM (2012). An atlas of the Epstein-Barr virus transcriptome and epigenome reveals host-virus regulatory interactions. Cell Host Microbe.

[26] Arvey A, Tempera I, Lieberman PM (2013). Interpreting the Epstein-Barr Virus (EBV) epigenome using high-throughput data. Viruses.

[27] Smith ZD, Meissner A (2013). DNA methylation: roles in mammalian development. Nat Rev Genet.

[28] Du J, Johnson LM, Jacobsen SE, Patel DJ (2015). DNA methylation pathways and their crosstalk with histone methylation. Nat Rev Mol Cell Biol.

[29] Schlesinger Y, Straussman R, Keshet I, Farkash S, Hecht M, Zimmerman J, Eden E, Yakhini Z, Ben-Shushan E, Reubinoff BE, Bergman Y, Simon I, Cedar H (2007). Polycomb-mediated methylation on Lys27 of histone H3 pre-marks genes for de novo methylation in cancer. Nat Genet.

[30] Ohm JE, McGarvey KM, Yu X, Cheng L, Schuebel KE, Cope L, Mohammad HP, Chen W, Daniel VC, Yu W, Berman DM, Jenuwein T, Pruitt K (2007). A stem cell-like chromatin pattern may predispose tumor suppressor genes to DNA hypermethylation and heritable silencing. Nat Genet.

[31] Widschwendter M, Fiegl H, Egle D, Mueller-Holzner E, Spizzo G, Marth C, Weisenberger DJ, Campan M, Young J, Jacobs I, Laird PW (2007). Epigenetic stem cell signature in cancer. Nat Genet.

[32] Laugesen A, Helin K (2014). Chromatin repressive complexes in stem cells, development, and cancer. Cell Stem Cell.

[33] Imai S, Nishikawa J, Takada K (1998). Cell-to-cell contact as an efficient mode of Epstein-Barr virus infection of diverse human epithelial cells. J Virol.

[34] Kaneda A, Fujita T, Anai M, Yamamoto S, Nagae G, Morikawa M, Tsuji S, Oshima M, Miyazono K, Aburatani H (2011). Activation of Bmp2-Smad1 signal and its regulation by coordinated alteration of H3K27 trimethylation in Ras-induced senescence. PLoS Genet.

[35] Kaneda A, Nonaka A, Fujita T, Yamanaka R, Fujimoto M, Miyazono K, Aburatani H (2016). Epigenomic Regulation of Smad1 Signaling During Cellular Senescence Induced by Ras Activation. Methods Mol Biol.

[36] Denny SK, Yang D, Chuang CH, Brady JJ, Lim JS, Gruner BM, Chiou SH, Schep AN, Baral J, Hamard C, Antoine M, Wislez M, Kong CS (2016). Nfib Promotes Metastasis through a Widespread Increase in Chromatin Accessibility. Cell.

[37] Shen L, Shao N, Liu X, Nestler E (2014). ngs.plot: quick mining and visualization of next-generation sequencing data by integrating genomic databases. BMC Genomics.

