# Supplementary material for: Histone modification alteration coordinated with acquisition of promoter DNA methylation during Epstein-Barr virus infection
Source: Oncotarget. 2017 Jul 21;8(33):55265–79. doi: 10.18632/oncotarget.19423 (PMC5589657; doi:10.18632/oncotarget.19423)
Supplement: Supplementary file 1 [file oncotarget-08-55265-s001.pdf]

Histone modification alteration coordinated with acquisition of promoter DNA methylation during Epstein-Barr virus infection

SUPPLEMENTARY MATERIALS

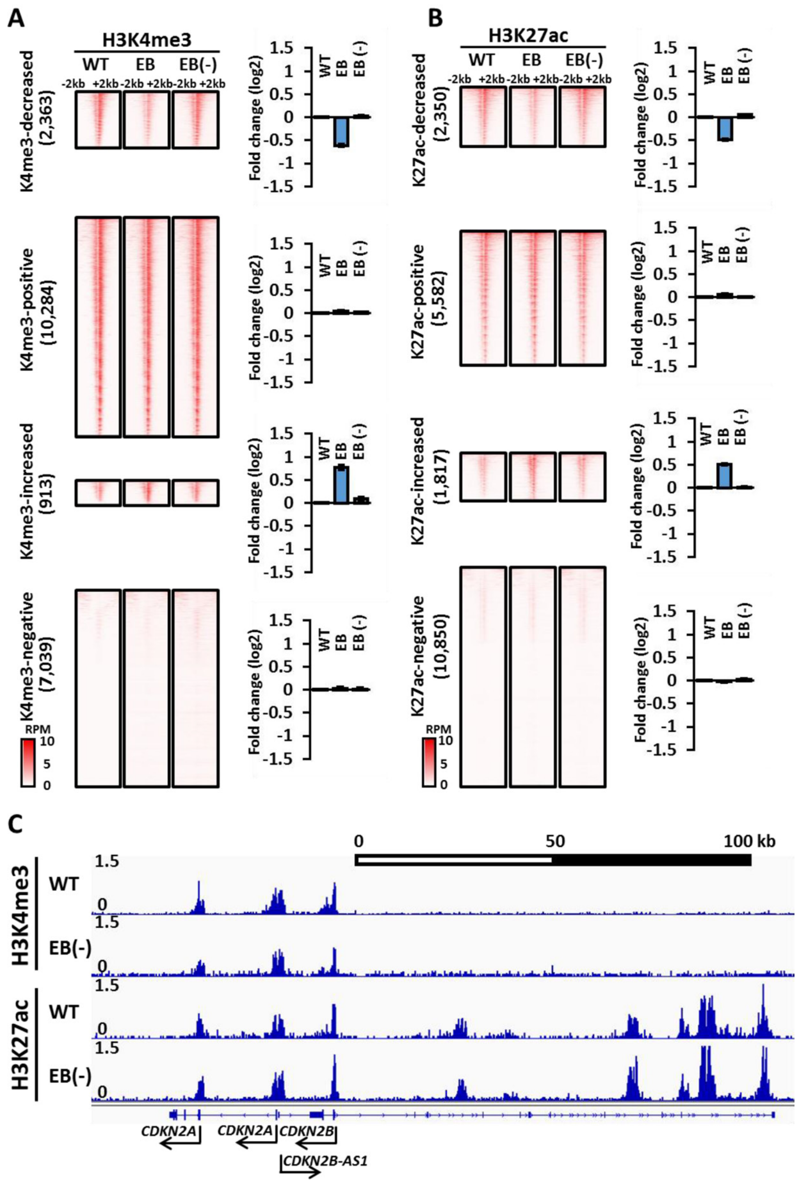

Supplementary Figure 1: Histone modification in control MKN7 cells cultured for 18 weeks without EBV infection. H3K4me3 and H3K27ac were analyzed using MKN7_EB(−) cells, and compared with MKN7_WT and MKN7_EB_A1 cells. *WT*, MKN7_WT cells. *EB*, MKN7_EB_A1 cells. *EB(−)*, MKN7_EB(−) cells. (**A**) H3K4me3 signals around promoters. While decrease and increase of H3K4me3 signals were observed in as many as 2,363 genes and 913 genes in MKN7_EB_A1 cells (*See* also Figure 4), they were generally not observed in MKN7_EB(−) cells. While marked downregulation of "decreased" genes (*top*) and upregulation of "increased" genes (*third*) were observed in MKN7_EB_A1 cells, and gene expression was generally not altered in MKN7_EB(-) cells. (**B**) H3K27ac signals around promoters. While decrease and increase of H3K27ac signal were observed in as many as 2,350 genes and 1,817 genes in MKN7_EB_A1 cells (*See* also Figure 5), they were generally not observed in MKN7_EB(-) cells. While marked downregulation of "decreased" genes (*top*) and upregulation of "increased" genes (*third*) were observed in MKN7_EB_A1 cells, and gene expression was generally not altered in MKN7_EB(-) cells. (**C**) Distribution of ChIP-seq reads of representative region is shown. Compared with MKN7_WT cells, H3K4me3 and H3K27ac signals were generally not altered in MKN7_EB(−) cells.

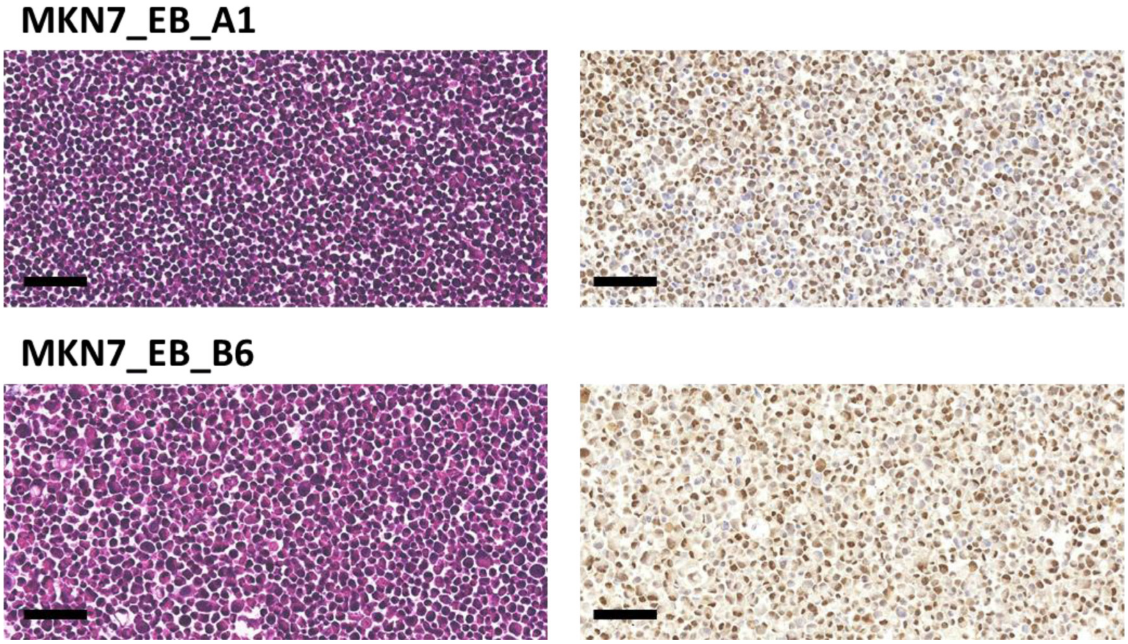

Supplementary Figure 2: EBER in situ hybridization. EBV existence in EBV-infected MKN7 clones was confirmed by EBER in situ hybridization. *Scale bar*, 100 µm.
